# Communities in restorative justice: insights from Colombia’s Special Jurisdiction for Peace

**DOI:** 10.3389/fsoc.2026.1936377

**Published:** 2026-09-24

**Authors:** Lorena Cecilia Vega Dueñas, Juliette Vargas, Laura Ordóñez-Vargas

**Affiliations:** 1 Faculty of Legal Sciences, Pontificia Universidad Javeriana, Bogotá, Colombia; 2 Colombian-German Institute for Peace (CAPAZ), Bogotá, Colombia; 3 KU Leuven, Leuven, Belgium; 4 School of Social, Political, and International Studies, Universidad del Rosario, Bogotá, Colombia

**Keywords:** communities, ethnic groups, restorative justice, Special Jurisdiction for Peace, transitional justice

## Abstract

While restorative justice recognises communities and victims as distinct key stakeholders, important questions remain about how these categories should be distinguished and why community participation is necessary, particularly in transitional justice contexts where systematic violence may directly affect entire human groups and territories. This article addresses these questions through a conceptual analysis of Colombia’s Special Jurisdiction for Peace (JEP), focusing on Macrocase 05. It argues that, in transitional settings, the distinction between communities and collective subjects recognised as victims becomes blurred, thereby challenging the differentiated roles traditionally assigned to victims, offenders and communities in restorative justice models developed for interpersonal conflicts. Far from constituting a conceptual inconsistency, this convergence is a defining feature of the JEP’s restorative model and contributes to broadening the conventional understanding of the role of communities in restorative justice.

## 1 Introduction

Restorative justice is a justice paradigm that shifts the focus from punishment to repairing harm, ensuring accountability, and promoting the active participation of those affected (United Nations Office on Drugs and Crime, 2020). Through dialogue-based processes involving victims, offenders, and communities, it seeks to repair harm and rebuild social relationships. In transitional justice settings, however, its implementation faces particular challenges due to the scale of violations, the collective nature of many harms, and the need to reconcile truth, justice, reparation, and guarantees of non-repetition (Weitekamp et al., 2006).

In Colombia, restorative justice is a guiding principle of the Special Jurisdiction for Peace (JEP), established by the 2016 Final Peace Agreement as the judicial component of the Comprehensive System for Peace. Its mandate is to investigate, clarify, prosecute, and sanction serious human rights and international humanitarian law (IHL) violations committed during the armed conflict, placing victims at the center and promoting their effective participation. The JEP was conceived as an exceptional and temporary jurisdiction, initially established for a period of fifteen years, extendable for an additional five years (Government of Colombia Revolutionary Armed Forces of Colombia–People’s Army (FARC-EP), 2016, p. 143 et seq).

Restorative justice traditionally brings together three key stakeholders: victims, offenders, and the community. Yet, as Fonseca Rosenblatt (2015) observes, despite broad recognition of the community’s importance, there is limited conceptual clarity about who constitutes the community and what justifies its participation in restorative processes. This question becomes particularly significant in transitional justice, where the harms of violence extend beyond individual victims to affected communities, collective identities, and territories. In such contexts, the community is not merely a passive recipient of restorative outcomes but a key stakeholder in processes of accountability, reparation, and the reintegration of perpetrators.

Building on this observation, this article asks two research questions: how are the place and role of communities shaped and redefined within restorative justice in transitional contexts? What implications does this have for the conceptual and empirical discussion of community participation in restorative processes? Based on these questions, the article argues that, in transitional settings, the categories of victims and communities tend to overlap. The same collective may constitute the social environment affected by violence, participate as an actor in the restorative process and, at the same time, be recognized as a collective subject of victimization and reparation. This lack of a clear delineation between the two categories, which constitutes a structural characteristic of the restorative processes developed by the JEPs territorial macrocases, does not necessarily represent a conceptual deficiency; on the contrary, it raises important implications for the identification of the subjects called upon to participate, the representation of collective interests, and the definition of reparation measures.

In this sense, this research is relevant because it makes it possible to question the existing conceptual delineations concerning the actors of restorative justice and to contribute to the discussion of the role of communities in restorative settings, considering the particularities and contributions offered by a particular and situated transitional model and context.

Theoretically, this analysis is situated within an interdisciplinary dialogue among legal anthropology, theories of restorative and transitional justice, conflict and peace studies, and decolonial feminist theories. Legal anthropology contributes an understanding of law as a social, historical, and cultural phenomenon shaped by power relations. It also provides analytical tools for examining the intersections between the legal and social spheres, the interactions among different normative systems, and the ways in which justice is materialized in concrete lived experiences (Krotz, 2002; Nader, 2002; Merry, 1988; Starr and Collier, 1989).

At the same time, the concept of community adopted by the JEP resonates with decolonial feminist perspectives by recognizing communities as collective victims and rights-bearing subjects. From this perspective, communities are understood not as a mere aggregation of individuals but as political and historical subjects whose racialized and gendered existence is deeply intertwined with territory and nature (Lozano Lerma, 2010; Gargallo, 2014; Paredes, 2017; Segato, 2011). Furthermore, through its commitment to legal pluralism and interjurisdictional dialogue, the JEP acknowledges the autonomy of Indigenous and other ethnic peoples as well as the legitimacy of their own justice systems (Santos, 2001; Sierra, 2011), while also incorporating an intersectional approach to the differentiated impacts of armed conflict (Viveros Vigoya, 2016).

In line with the interpretive approach, this analysis is based on a qualitative methodology, particularly, a critical review of the specialized literature and on the analysis of public sources, including legislation, case law, official documents of the JEP, and the Public Hearing for the Acknowledgment of Truth and Responsibility in Macrocase 05, which is used as an illustrative reference for the Colombian transitional justice model.

This case is selected because it provides a particularly suitable setting for examining the relationship between communities and collectively recognized victims. In this case, the recognition of Indigenous peoples and Afro-descendant communities as collective victims, together with the collective harms inflicted on their territory, cultural identity, forms of organization, and social fabric, demonstrates how these two categories become integrated within the JEP’s restorative model. Furthermore, among the JEP cases that involve collective victims, Macrocase 05 is one of the most advanced cases in procedural terms so it allows an analysis of the role of communities in a crucial procedural stage, the hearing of acknowledgement of responsibility, as will be explained.

The article is structured into three chapters. The first presents the theoretical framework on restorative justice, communities, and the main challenges of this paradigm in transitional settings. The second examines the incorporation of restorative justice within the JEP and the role of communities in this context. The third analyzes JEP Macrocase 05, focusing on the communities involved, the overlap between communities and collective victims, and how this overlap is reflected during the Public Hearing for the Acknowledgment of Truth and Responsibility. The article concludes by summarizing the main findings and discussing their implications for restorative justice theory and practice in transitional settings.

## 2 Restorative justice in transitional settings: conceptual foundations

### 2.1 Defining restorative justice: communities as key stakeholders

Restorative justice emerged in Canada in the 1970s (McCold and Wachtel, 2003) and has been described as an elastic and multidimensional concept (Walklate, 2016), reflecting the absence of a universally accepted definition. This conceptual ambiguity has led some scholars to warn against its “misappropriation,” whereby restorative justice is equated with any approach that departs from traditional justice or emphasizes victims’ interests (Clamp, 2016).

One of the most widely accepted definitions is that proposed by Johnstone and Van Ness (2007), according to which restorative justice revolves around three core elements: the encounter among all stakeholders in the conflict—victims, offenders, and members of the community; the reparation of the harm caused by crime; and the transformation of relationships among individuals and of social life (Van Ness and Heetderks Strong, 2015).

Encounter promotes the active participation of victims, offenders, and communities in discussing the offense, responsibilities, the harm caused, the needs arising from the conflict, and possible forms of reparation (Derby, 2021), recognizing communities as a catalyst for the restorative process (Ordóñez and Brito, 2004). Reparation seeks to restore the harm suffered by victims and communities, as far as possible through the offenders themselves (Derby, 2021), thereby contributing to the reconstruction of social relationships and collective trust. To this end, it is essential to guarantee the effective participation of victims (Suchkova, 2011), as it provides material, practical, and symbolic meaning to reparation (Marín Ortiz, 2025). Finally, transformation seeks to generate positive change in victims and offenders by strengthening reintegration, and in communities by fostering a sense of belonging, security, and community participation, among other outcomes (Derby, 2021). In this context, Braithwaite’s (1989, 2002) theory of reintegrative shaming maintains that punishment should produce shame in the offender while at the same time promoting their reintegration (Vega Dueñas and Hernández-Jiménez, 2025).

Regardless of the definition adopted, restorative justice represents a paradigm shift from traditional justice by understanding crime as harm inflicted on people rather than merely an offence against the State, thereby prioritizing the repair of that harm (Zehr, 1990). Consequently, instead of focusing primarily on punishment, restorative justice emphasizes the harms and needs of victims, the responsibilities of offenders, and the participation of all those with a legitimate interest in the offence and its reparation (Zehr, 2015).

Restorative justice is also flexible in terms of its role and timing. It may operate through different models—full, dual, backup, or hybrid (Steinl, 2017)—and can be implemented outside the criminal justice system or at different stages of the criminal process, from investigation to post-release (Derby, 2021). Above all, restorative justice is a perspective on justice (Vega Dueñas and Parmentier, 2024), grounded in humanistic values such as recognition, reparation, respect, voluntariness, inclusion, empowerment, safety, accountability, and transformation (United Nations Office on Drugs and Crime, 2020).

Restorative justice promotes the participation and commitment of victims, offenders, and communities as the primary stakeholders in crime and its restoration (Zehr, 2015). Based on this premise, McCold and Wachtel (2003) distinguish between fully, mostly, and partly restorative practices, depending on whether all three parties, two of them, or only one is involved.

Regarding victims, this approach seeks to recognize and repair the harm they have sufferedand toaddress their needs for information and participation while providing opportunity for communication (Walgrave, 2011) expression, empowerment, and reparation by offenders (Zehr, 2015). Regarding offenders, restorative justice encourages them to acknowledge responsibility for their actions, understand their consequences, contribute to the reparation of victims, and promote their reintegration into society (Zehr, 2015), without excluding imprisonment when it is considered necessary (Steinl, 2017). Finally, crime also affects communities and, accordingly, restorative justice promotes the participation of community members in resolving the conflict, taking into account their needs, roles, and responsibilities (Zehr, 2015).

Although there is broad consensus regarding who qualifies as a victim and who qualifies as an offender, even though situations of mass violence may also give rise to particular dynamics such as “role reversal” (Friday, 2009), no comparable agreement exists regarding the concept of community (Fonseca Rosenblatt, 2015). It is therefore necessary to examine the main contributions of literature concerning who constitutes the community and why communities are important in restorative justice.

On the one hand, broadly speaking, the community includes any person with an interest in the resolution of a criminal conflict who chooses to participate (Bazemore and Umbreit, 2001). It has also been distinguished between primary stakeholders, who have close personal ties to victims or offenders, and secondary stakeholders, who are affected more indirectly, such as organizations, public officials, or society as a whole (McCold and Wachtel, 2003). More specifically, Van Ness and Heetderks Strong (2015) distinguish three understandings of community: geographical communities, communities of care or interest, and society as a whole. Similarly, Clamp (2014) differentiates between micro-communities, comprising those directly affected by the offence, and macro-communities, encompassing society at large and national institutions.

On the other hand, communities are essential because their support and participation contribute to repairing victims, holding offenders accountable, and facilitating the reintegration of both into society (Pranis, 1995; Guardiola Lago and Tamarit Sumalla, 2013). As the social context in which crime occurs (Britto Ruiz, 2006), communities also help sustain restorative agreements, strengthen relationships and trust, and support the long-term restoration of social cohesion (Pranis, 1995).

Although the importance of communities in restorative justice is widely recognized, their participation raises several challenges, including ensuring meaningful and representative participation, defining who speaks on behalf of the community, avoiding tokenistic involvement and the professionalization of community representatives, and preventing excessive influence that may lead to disproportionate sanctions or the exclusion of offenders (Rossner and Bruce, 2016). If restorative values are not properly upheld, these dynamics may even produce outcomes that are more punitive than retributive justice (Roche, 2007).

### 2.2 Restorative justice in transitional contexts: scope and challenges

Restorative justice may be applied in a wide range of contexts, from the management of school conflicts to the prosecution of international crimes. This article focuses on the application of restorative justice within the framework of transitional justice, understood as justice adapted to societies transforming themselves after a period of systematic or widespread human rights violations (International Center for Transitional Justice, 2009). Accordingly, this study is situated within a field in which the application of restorative justice has traditionally been the exception rather than the rule. This is due, among other factors, to the seriousness and complexity of human rights violations and mass crimes that constitute the subject matter of transitional justice mechanisms (Parmentier, 2011).

The application of restorative justice in transitional contexts gained special relevance, particularly with South Africa’s Truth and Reconciliation Commission and Rwanda’s Gacaca Tribunals (Tutu, 1999; Clark, 2010). Despite their significance as transitional justice models, both initiatives have been subject to criticism. Scholars have argued that restorative justice played only a limited role within the Truth and Reconciliation Commission (Gade, 2013), while the Gacaca Courts revealed the difficulties of achieving the effective reintegration of offenders into communities still marked by trauma and mistrust, despite contributing to truth-seeking and accountability (Musengimana, 2024).

More broadly, the application of restorative justice in transitional settings has expanded traditional conceptions of justice while also revealing important challenges. It calls for a holistic approach that complements retributive justice with restorative and other context-specific forms of justice, aiming not only to address past human rights violations but also to transform the structural causes of violence, strengthen communities, promote peacebuilding, and prevent future harm (Nickson and Braithwaite, 2014; Sullivan and Tifft, 2001). At the same time, the scale and gravity of international crimes make it impossible to rely on traditional case-by-case models of reparation. Instead, restorative justice in transitional settings requires a macro-level approach that recognizes the collective nature of many harms and the diversity of victims’ and communities’ understandings of reparation, shaped by their cultural, religious, and political contexts. Within this framework, restorative justice promotes dialogue and the participatory design of reparative measures that are responsive to these diverse experiences rather than imposing a single model of reparation (Clamp, 2016; Llewellyn and Howse, 1999).

In transitional contexts, it has been recognized the crucial role of communities to develop relational forms of reconciliation and responsibility that address the structural character of harm, and challenge individual accountability (Harbin and Llewellyn, 2016). However, the place and role of communities that may overlap with victim as stakeholders has not been analyzed under the light of the practical and theoretical implications for restorative justice. Transformative justice scholars, provide though important insights on participation and local appropriation in transitional justice that may be useful to understand the possibilities, challenges and shortcomings when it is about restorative justice practices and processes in transitional settings. Thus, (i) the contours of the category of victim or perpetrator could be problematic when among the collective, some individuals identify differently or may had a disruptive or contentious role: (ii) Existing power dynamics and social inequalities may reproduced within the collective; (iii) Diverse views and interests coexist within the collective and this raises issues of representation and legitimacy of spokespersons; and finally (iv) while the inclusion of communities require large-scale participation and consequently requires mote time transitional justice is designed to be time restrictive (Firchow and Selim, 2022). As it will be explained further, the Colombian experience faces to some extent all these challenges, but at the same time provides the opportunity of strengthening existing organizational processes.

## 3 Restorative justice in the Colombian transitional justice system: the place of communities

### 3.1 Restorative justice as the guiding paradigm of the Special Jurisdiction for Peace (JEP)

The JEP, as the judicial component of Colombia’s Comprehensive System for Peace (SIP), is grounded in a restorative paradigm that seeks to repair harm, restore victims’ dignity, and rebuild the social fabric (Government of Colombia Revolutionary Armed Forces of Colombia–People’s Army (FARC-EP), 2016, p. 144; Greve and Vega Dueñas, 2023). This represents a shift from a predominantly retributive model toward a relational, forward-looking, and transformative conception of justice.

This shift is distinctive in the Colombian context because it has been institutionalized within a criminal tribunal. Although the JEP retains the State’s punitive authority, it combines retributive and restorative logics, constituting a hybrid model (Steinl, 2017). This hybrid character is reflected in its system of sanctions, which depends on offenders’ acknowledgment of responsibility and contribution to the truth^1^. Such acknowledgement is central to the reintegration of perpetrators into society and affected communities. Through public hearings and other symbolic acts, perpetrators are expected to recognise their responsibility, acknowledge the harms caused, and commit to reparative measures. Following Braithwaite’s concept of reintegrative shaming (1989), this process seeks not to stigmatise or exclude, but to link accountability with reparation for victims, communities, and territories.

Accordingly, restorative justice is not limited to a complementary mechanism or a specific stage of the proceedings (Derby, 2021). Instead, it permeates the various procedural stages and proceedings before the JEP, expanding the judicial process to incorporate symbolic, social, and relational dimensions traditionally considered beyond the domain of law.

This configuration reflects the JEP’s structural approach to the armed conflict. By prioritizing patterns of macro-criminality and macro-victimization, and focusing on the most serious and representative crimes, territories, and those most responsible^2^, the JEP seeks to understand violence as a systematic phenomenon rather than a collection of isolated events. Accordingly, it has prioritized eleven macrocases^3^ based primarily on two criteria: specific patterns of victimization and territories historically and systematically affected by the armed conflict.

Consistent with this structural approach to the conflict, the JEP also incorporates, as cross-cutting perspectives, a territorial approach together with differentiated and intersectional approaches (including gender, ethnic-racial, age, and disability perspectives)^4^, which make it possible to recognize the specific ways in which different population groups and territories have experienced multiple forms of violence (Special Jurisdiction for Peace, 2020). These approaches do not operate merely as descriptive categories; rather, they directly and transversally shape the definition of harm, the modalities of participation, and the forms of reparation. Additionally, recognizing territories and nature as victims also reflects the biocentric turn that the JEP is bringing to transitional justice with a restorative approach (Ordóñez-Vargas et al., 2023).

Colombia represents one of the most advanced examples of the institutionalization of restorative justice within a transitional justice framework. This model constitutes both an institutional and jurisprudential innovation (Constitutional Court of Colombia, 2018a, 2018b, 2019) and raises important questions about the compatibility -or incompatibility- between restorative justice and the State’s duty to impose sanctions. Aware of these challenges, the JEP has developed a conceptual framework, proposing the concept of restorative transitional justice and defining it as: “a form of participatory, collaborative, and forward-looking justice that seeks to address the harms and impacts caused in the context of the armed conflict, reintegrate the parties into society, and restore the social fabric through processes of acknowledgment and accountability, contributions to comprehensive reparation and the restoration of dignity, as well as measures aimed at prevention, protection, and guarantees of non-repetition” (Special Jurisdiction for Peace, 2024a, 2024b, p. 47). This definition will be used throughout this paper, while recognizing that it is a conceptual category arising from the innovations of the Colombian transitional justice model and that its scope remains under development and subject to ongoing debate.

### 3.2 Integration of communities with the collective subjects recognized as victims before the Special Jurisdiction for Peace (JEP)

It is important to recognize that the role of communities within the JEP can only be understood in light of the nature and scale of the Colombian armed conflict. Systematic patterns of victimization affected individuals, collective subjects, and territories simultaneously, with Indigenous and Afro-descendant peoples experiencing a historical continuum of violence, discrimination, and racism that was further intensified by the conflict. At the same time, these communities have resisted violence collectively through the defense of their cultures, territories, and systems of self-government^5^. This perspective is central to restorative justice in transitional settings, where communities are not merely stakeholders or the social context in which conflict occurs, but may themselves be subjects of collective harm and restoration.

On the one hand, how are the place and role of communities shaped and redefined within the JEP? Different groups have been identified as performing relevant community roles within JEP proceedings. These include the families and other support networks of victims and offenders; territorial leaders and civil society organizations that have not been directly victimized; communities located in areas of reincorporation or return to civilian life, as well as territories where development Programs with a territorial focus (PDETs)^6^ are being implemented; and communities in which restorative projects are carried out. Institutional actors are likewise recognized as part of the community, as they not only perform supervisory and facilitative functions within restorative processes but also actively contribute to the establishment of an effective justice process (Special Jurisdiction for Peace, 2024a, 2024b, pp. 100–101). The communities recognized by the JEP are consistent with Clamp (2014) distinction between the micro-community and the macro-community. However, communities have been also recognized by the JEP as victims themselves, when they share the same territory, cosmovision, common goals and/or life project and it has been directly affected by the armed conflict.

This article argues that in restorative scenarios in transitional settings, the boundaries between communities and collective subjects recognized as victims tend to blur, particularly between communities and the collective subjects recognized as victims. This overlap should not be understood as a conceptual problem requiring resolution but rather as a constitutive feature of the model. The JEP itself has acknowledged this (Special Jurisdiction for Peace, 2024a, 2024b, p. 98), emphasizing that the integration of these two roles is essential for understanding and addressing the complexities of this form of justice and its processes of reparation and reconciliation. Moreover, this overlap does not preclude characterizing the practices developed within the JEP as fully restorative, in accordance with the classification proposed by McCold and Wachtel (2003), insofar as they involve the participation of victims, offenders, and community representatives.

One of the factors that has facilitated the integration of the roles of victims and communities has been the expansion of the concept of victim by the JEP. Consistent with the principles of the centrality of victims and their comprehensive participation, the Jurisdiction recognizes as victims: individual victims, collective subjects, indirect victims, and even non-human entities such as territory and nature. Victims may obtain formal recognition before the Jurisdiction and acquire the status of *special interveners*, which enables them to participate actively and in multiple ways throughout the different stages of the proceedings (Special Jurisdiction for Peace, 2024b, p. 77).

It is important to emphasize that, for the JEP, the recognition of collective victims does not come at the expense of individual victims; on the contrary, it entails an expansion of the liberal conception of rights, which traditionally focuses exclusively on individuals, whether as victims or perpetrators, rather than on groups. It is also worth noting that not all collective victims are ethnic groups. Political and social organizations are also recognized as collective victims^7^. As of March 2026, the JEP had accredited 14,779 individual victims and 424 collective subjects (Special Jurisdiction for Peace, 2026), reflecting both the centrality of victims in its proceedings and the scale of victimization represented by these numbers, although they do not capture the total number of victims.

On the other hand, what implications does the participation of communities within the framework of the JEP have for the conceptual and empirical discussion? Thus, first, communities actively participate in judicial proceedings by intervening at different procedural stages and contributing to initiatives related to memory, truth, and guarantees of non-repetition, thereby extending justice beyond the courtroom. Second, they act as co-creators of reparation by participating in the design and implementation of restorative projects addressing territorial, cultural, and symbolic harms. Finally, communities provide emotional and social support to both victims and participants appearing before the JEP, facilitating restorative encounters and contributing to the rebuilding of social relationships, a role that is central to the restorative dimension of the process.

In this way, communities function as collective rights holders and political and historical actors in the restorative process, whose participation is indispensable to achieving the objectives of truth, reparation, and guarantees of non-repetition. However, this central role also gives rise to tensions. The incorporation of communities into a formal judicial framework raises questions concerning the limits of their participation, issues of representativeness, the risk of instrumentalization, and the power asymmetries inherent in the legal field (Vargas Trujillo, 2022).

Regarding communities and victims’ participation and representation before the JEP, it is predominantly collective, channeled through common legal representatives and spokespersons in order “to bring a degree of efficiency to the judicial process and to unite a movement for redress that may share many common elements, thereby facilitating collective representation of the traumatic past” (Special Jurisdiction for Peace, 2019, para. 111). However, even victims who do not belong to Indigenous or Afro-descendant communities generally participate collectively, while individual participation is reserved for exceptional cases involving privacy or other specific concerns. The JEP’s collective participation model therefore seeks not only to improve procedural efficiency, but also to strengthen existing organisational processes and foster new ones promoting social cohesion.

Within the JEP’s procedure involving acknowledgment of truth and responsibility^8^, victims may submit reports (Vega Dueñas, 2020), participate at different procedural stages, present observations, take part in public hearings, and contribute to the design of the *sanción propia* (own sanction) (Special Jurisdiction for Peace, 2024b, pp. 158 et seq.). Beyond their procedural role, victims actively question those appearing before the JEP, demand explanations, and contribute to decisions on sanctions and reparative measures, thereby shaping the construction of judicial truth. In this sense, victims and communities within this category act as co-producers of legal knowledge about the armed conflict. Nevertheless, despite the progress achieved and the good practices identified, several challenges remain regarding victim participation. Among the most significant are the insufficient provision of clear and timely information and the disparity in the opportunities for and forms of participation, which vary depending on the case or the chamber responsible (Valencia Cortés and Molano Ayala, 2023).

In the case of collective subjects recognized as victims, this position acquires an additional dimension. The recognition of socio-cultural, territorial, and spiritual harms, particularly in relation to Indigenous Peoples and Rrom, Black, Afro-Colombian, Raizal, and Palenquero communities, reflects an opening of the legal field to conceptions of harm that transcend the traditional legal categories centered on individuals rather than collective subjects. On the basis of the legal pluralism enshrined in the Colombian Constitution (Republic of Colombia, 1991, Articles 1, 7, and 246) and the recognition in the Final Peace Agreement that ethnic peoples have historically experienced conditions of injustice (Government of Colombia Revolutionary Armed Forces of Colombia–People’s Army (FARC-EP), 2016, p. 144), the JEP has established mechanisms for intercultural and inter-jurisdictional dialogue^9^ and recognizes Indigenous and other traditional systems of justice. This recognition is not merely symbolic; it legitimizes the coexistence of different normative systems and forms of justice while recognizing Indigenous and ethnic peoples as active participants in shaping understandings of harm, responsibility, and reparation.

## 4 Macrocase 05 of the Special Jurisdiction for Peace (JEP): the communities in context

### 4.1 Integration of communities and collective subjects recognized as victims in the context of Macrocase 05

Macrocase 05 will serve as the basis for examining the implementation of the “integrated concept” of communities and victims. The JEP opened Macrocase 05 on 8 November 2018 to investigate serious human rights violations and grave breaches of international humanitarian law committed between 1 January 1993 and 1 December 2016 by members of the State security forces and the FARC-EP guerrilla in two regions of south-western Colombia: northern Cauca and southern Valle del Cauca. Among the cases before the JEP, Macrocase 05 has the highest number of collective indigenous and afro-descendant victims while also includes peasant organisations and individual victims. Additionally, in contrast with other territorial cases, the hearings of acknowledgment of Macrocase 05 took place already in November and December 2025, so it allows us to analyse better this particular procedural stage under the lens of the role of communities as victims.

On the one hand, how are the place and role of communities shaped and redefined within the framework of Macrocase 05? Northern Cauca and southern Valle del Cauca have been historically affected by the armed conflict, with all armed actors contesting territorial and socio-economic control. These regions are also home to particularly vulnerable populations, including Indigenous Peoples, Afro-descendant communities, and peasant communities^10^. Indigenous peoples account for approximately 60.1% of the population of northern Cauca, and the presence of Afro-descendant communities is also particularly significant in several municipalities in the region (National Administrative Department of Statistics (DANE) and Universidad del Valle, 2010, p. 35 y 77).

Given the strong presence of ethnic communities in these regions, they may be understood as intercultural territories in which different collective identities, worldviews, cultures and knowledge systems converge. Accordingly, Macrocase 05 focuses not only on individual members of ethnic communities, but also on those communities as collective subjects. This has significant implications for understanding the multiple layers of harm caused by the conflict, ranging from individual, familial and communal harm to harm inflicted upon the territory itself. Likewise, the organisational forms of Indigenous and Afro-descendant peoples have shaped the contours of who is recognised as a victim before the JEP and the extent to which that concept overlaps between victims and communities as collective subjects.

Approximately 166 collective subjects and 316 individuals have been accredited as participating victims in Macrocase 05. These collective subjects include 45 Indigenous peoples, 88 community councils^11^, 8 peasant organisations and 25 other victims’ organisations. In addition, around 180.000 individuals are members of these collective subjects. Macrocase 05 is therefore the macrocase with the highest number of collective subjects and participating victims within the Special Jurisdiction for Peace (2025a, 2025b).

Among the collective subjects recognized in Macrocase 05, indigenous peoples are represented primarily by the Cauca Regional Indigenous Council (CRIC), which coordinates its work with organizations such as the Cauca Regional Indigenous Organization (ORIVAC) and the Association of Indigenous Councils of Northern Cauca (ACIN)^12^. For their part, Afro-descendant communities are represented by organizations such as the Association of Community Councils of Northern Cauca (ACONC)^13^, as well as by other broadly representative community councils, such as the Community Council of the Cauca River Basin^14^.

On the other hand, what implications does community participation within the framework of Macrocase 05 have for the conceptual and empirical discussion? Communities in Macrocase 05 are important, as collective organizational forms and grassroots organizations. First, because they have contributed to the JEP by submitting collective reports and coordinating the legal representation of Indigenous resguardos and community councils through grassroots organizations such as CRIC, ORIVAC, and ACONC. Second, their participation has enabled the JEP to better understand the collective, intergenerational, and territorial dimensions of harm, recognizing that a single violation may affect an entire community rather than merely constituting an accumulation of individual harms. Thirdly, the involvement of Indigenous and ethnic communities has strengthened the recognition of their political authorities and promoted intercultural dialogue that respects their own systems of justice.

This article argues that in restorative scenarios in transitional settings, the boundaries between communities and collective subjects recognized as victims tend to blur, and this defining characteristic of the model can be observed in Macrocase 05. The overlap between victims and communities as stakeholders in JEP Macrocase 05 can be understood as the result of three interrelated factors. First, the armed conflict affected entire populations that share a common ethnic identity, territory, culture and knowledge system, or common goals and socio-political processes. Secondly, Indigenous and Afro-descendant peoples have long understood themselves as collective subjects; accordingly, harm caused to one or several members has a direct impact on the community itself. Thirdly, ethnic communities maintain profound relationships with their territories and ecosystems, which cannot be understood merely as geographical spaces or sources of natural resources. Ancestral territory is a living entity, and humans and more-than-human beings form part of it. Consequently, the identity of ethnic communities, their worldviews, spiritual and cultural practices, and livelihoods are deeply embedded in collective land. The harm caused by the armed conflict has therefore affected both these relationships and multiple dimensions of the life of the collective subject.

One of the elements that explains the integration of the roles of victims and communities is that the harm caused by the armed conflict to ethnic groups has multiple dimensions. The JEP has recognised that beyond the impact on individuals and their relatives, the conflict has produced collective harms in the case of ethnic communities:

*The presence of various armed groups in their territory has had a detrimental effect on their cultural identity, causing discord amongst the population, as their freedom of movement has been restricted, thereby limiting their self-determination and the exercise of governance, as well as their cultural use of spaces considered sacred to their individual and collective lives […].*^15^

Afro-descendant communities have identified harms associated with structural racism, the disruption of their daily lives and ancestral cultural practices, and the breakdown of their social fabric, resulting from conflict-related violence such as forced displacement, land dispossession, killings, enforced disappearances, child recruitment, illegal economies, and anti-personnel mines^16^.

Similarly, the Nasa^17^, Kokonuko, Misak, Inga, Totoró and Yanacona peoples agree that the conflict has affected their cultural integrity, their territories, and their political and organisational autonomy:

*Furthermore, attention has been drawn to the existence of collective harm to three fundamental values of the communities: cultural integrity ( ); the territory ( ); and political and organizational autonomy and integrity*^18^.

Moreover, ethnic communities have sought the recognition of ancestral territories and natural entities, such as the Cauca River, as victims of the armed conflict, reflecting their worldview and the special relationships they maintain with these living territories and ecosystems (Izquierdo Torres and Viaene, 2024).

The reaffirmation and development of their identities, cultures and knowledge systems are therefore inseparable from their ancestral territories.

*Our ancestral and sacred territory has suffered violations, alterations, desecrations, occupations and damage as a result of the internal armed conflict, which has negatively transformed the bond that indigenous communities had with their territory, undermining the balance, harmony and autonomy of the Nasa indigenous people of northern Cauca* ^19^ *.*

Similarly, for Afro-descendant peoples, cultural practices, histories of resistance and flourishing, and collective identities are deeply tied to territory. For many communities, rivers have become essential to communal and cultural life.

*“Firstly, the violence along the Cauca River and the cessation of activities centered on the river contributed to the destruction of community ties and the cultural identity of the communities, given that community social life took place around the river”.*^20^

### 4.2 Communities and victims before the JEP: the hearing of acknowledgement for Macrocase 05

Given the limited space available to provide a comprehensive description of the judicial context and the specific stage of the proceedings, we highlight certain elements from the hearings that illustrate this overlap between communities and victims, as well as its scope and limitations. To begin with, the Hearing of Acknowledgment of Truth and Responsibility^21^ for Macrocase 05, took place over 4 days, and collective subjects were widely represented through ethnic and peasant organizations accredited by the JEP. This representation is organized into three groups: indigenous communities^22^, Afro-Colombian communities^23^ and peasant communities^24^. Victims play a leading role in this hearing. Victims and their spokespersons have structured opportunities to speak; they present representative accounts with specific names and contextual details; and in at least one case (María Fernanda Arboleda), they break from the testimonial format to directly question the witnesses. Their participation is neither passive nor merely symbolic, but active. However, this leading role is structurally framed by the judiciary: victims have a voice within a predetermined format (10 min, a fixed order of speaking, and preselected representative cases). This relationship and tension between a leading role and institutional framing are constant.

From a legal standpoint, the organizations participate as accredited collective subjects and intervenors in the proceedings, with formal legal representation. Their testimonies are not merely moral statements but contributions to the drafting of the order that guides the findings. The judiciary explicitly acknowledges that Order No. 01 of 2023^25^ (Special Jurisdiction for Peace [JEP], Chamber for the Recognition of Truth, Responsibility and Determination of Facts and Conduct, 2023) was drafted using information provided by the victims and ethnic organizations, which grants the victims the status of co-architects of the process, not merely recipients.

The categories of collective subjects accredited as victims and communities are never distinguished during the hearing. The spokespersons speak interchangeably on behalf of both victims and communities without drawing a line between the two statuses. The same organizations are simultaneously identified as representatives of accredited victims and as self-governing community authorities.

This overlap is not a colloquial or imprecise usage, but rather the way in which the subjects themselves define and represent themselves. According to what they say, it is as if, for ethnic communities, being a victim of the conflict and being a community with a history, territory, and life project are inseparable dimensions. The harm they suffered was not harm to individuals but to their collective existence, which makes the category of “collective victim” and that of “affected community” one and the same, from their perspective.

*Today we represent here our peoples, our communities, all those who today cry out for peace, justice, and truth. (Afro-Colombian representative, harmonization session, 8:09)*

*I am here because I am a victim of the conflict—at the family, community, and territorial levels… it has affected our entire ancestral heritage, including our spiritual traditions, our territories, and our families. (Victim in institutional video, 3:40:44)*

As for opportunities for participation, victims intervene at three key moments: during the introduction of spokespersons at the beginning of the hearing, during the presentation of each case, and through prerecorded audiovisual statements shown during the recess. The only unstructured opportunity for participation is the direct questioning of María Fernanda Arboleda.

Ethnic authorities play a dual role in this hearing that is not clearly distinguished from the role of victims. Junior Elkin Pilcueé identifies himself as an “indigenous authority of the Corinto reservation” and acts as a spokesperson for the indigenous victims during the same session; Luz Jeni Quitumbo identifies herself as an “indigenous authority of the Cuesta Taquiwe Las Huacas Grand Council” and serves in the same capacity for the reservations in Valle. The Afro-Colombian spokesperson speaks from a position that simultaneously combines the status of victim and that of community authority with her own political representation.

This fusion of positions is the most direct manifestation of the overlap posited by the research hypothesis. Ethnic authorities do not appear first as authorities and then as representatives of victims, nor vice versa: they always speak from both positions at once, because for these communities, the status of authority and that of bearer of collective harm are inseparable. The opening remarks provide the clearest example: the representatives are simultaneously spiritual authorities, victims of the conflict, and community spokespersons in a single speech act.

Julián Gutiérrez (an authority from the Ques Yukiwe reservation in Florida) illustrates this duality in a particular way: he presents himself as an authority standing in for a direct victim who could not attend out of fear, thereby assuming the voice of that individual victim from his position of collective authority.

The harm is presented exclusively as collective. None of the ethnic authorities reduce the harm to the individual or family level: in all cases, the harm is described as an impact on the people, the territory, spirituality, the intergenerational transmission of knowledge, community autonomy, and indigenous justice systems.

*We request recognition that the actions of the FARC’s Arenas and Gabriel Galvis mobile columns have caused profound and multidimensional harm to Afro-Colombian communities. These acts constitute crimes against humanity, as they were not only motivated by economic reasons but also disrupted daily life and dismantled ancestral cultural, spiritual, and medicinal practices by preventing the transmission of knowledge and access to sacred sites. (Erley Ibarra, 1:57:24).*

Territory is the most prominent and recurring theme in all the statements by victims and ethnic representatives. This is not a matter of geographical impacts or the loss of material goods, but rather the rupture of the ontological relationship between the communities and the territory: the territory as the condition of possibility for life, as a sacred entity, as a repository of collective memory, and as a space for the exercise of autonomy. The territorial impacts included confinement, restrictions on agricultural, fishing, and gathering activities, blocked ancestral routes and rivers, abandonment and militarization of land, violations of collective territorial rights, restricted access to cemeteries, and landmines in sacred areas that disrupted spiritual practices.

Regarding acceptance of the recognition, the ethnic authorities do not reject the process, but neither are they satisfied with a mere formal acceptance. The prevailing stance is one of active demand: Junior Pilcueé calls on the national government; the Afro-Colombian spokesperson demands that recognition encompass the destroyed ancestral cultural, spiritual, and medicinal practices; Magnolia Velasco Meneces demands full and detailed truth regarding landmine policies, direct participation in humanitarian demining, concrete reparations (not merely symbolic ones), and real guarantees of non-repetition.

*Forgiveness is not demanded; it is built, and it can only emerge when the truth is complete, when reparations are genuine, and when you walk alongside the communities to heal what was destroyed. (Magnolia Velasco, 6:16:27)*

During the hearing, the magistrate uses the terms collective victims and communities interchangeably when referring to Indigenous, Afro-Colombian, and peasant groups affected by the conflict, suggesting that this overlap is embedded not only in community discourse but also in the JEP’s institutional language. The recognition of the Cauca River and Nasa territory as rights-bearing subjects further expands the range of entities protected within its restorative framework.

## 5 Discussion

This article examined how the place and role of communities are configured and redefined within the framework of restorative justice in a transitional context such as that of the JEP, particularly in territorial macrocases and, more specifically, in Macrocase 05. Based on an analysis of institutional discourse, case law, and Truth Acknowledgment Hearings, it showed that the categories of community and collective victim tend to overlap and, at times, become indistinguishable within the JEP’s restorative model.

This finding invites a reconsideration of one of the central debates in the classical theory of restorative justice. Rather than reflecting an ambiguity regarding who constitutes the community, the experience of the JEP demonstrates a simultaneous expansion and reconfiguration of the categories of victim and community by recognizing collective victims as procedural subjects and central actors both in judicial proceedings and in the broader social processes articulated around them. In this sense, the community is no longer necessarily a third party external to the conflict and may, in certain contexts, become an affected subject, an active participant, and a beneficiary of reparation.

The JEP recognizes that mass violence produces not only individual but also collective, territorial, cultural, spiritual, and organizational harms, positioning communities as political, legal, and historical subjects central to memory, reparation, and peacebuilding. Drawing on decolonial approaches, it has further expanded the recognition of collective victims to include ethnic peoples, organizations, territories, and even nature. Likewise, the recognition of Indigenous justice systems, NARP authorities, and Rrom institutions reflect a pluralist conception of law that challenges a single universal legal order, while interjurisdictional and intercultural mechanisms enable communities to participate in the construction of restorative processes and outcomes.

Overall, the experience of the JEP contributes to broadening the restorative justice paradigm by adapting it to contexts of macro-criminality and mass victimization, while incorporating territorial, differential, and intersectional approaches. The convergence or overlap between community and victim challenges the classical conception of the community as a third party external to the conflict and suggests that, in certain transitional contexts, the two categories may coincide. This shift makes it possible to rethink the traditional categories of restorative justice and their capacity to address the challenges arising from collective and large-scale violence.

From a comparative perspective, the JEP constitutes a particularly relevant case of the judicial institutionalization of restorative principles in a transitional context. Its experience therefore represents not only an institutional and jurisprudential innovation, but also a theoretical challenge for scholarship on restorative justice, transitional justice, and peacebuilding.

## Footnotes

1 The JEP imposes three types of sanctions, considering the contribution to the truth and the acknowledgment of responsibility: ordinary, alternative and own sanctions (Congress of the Republic of Colombia, 2019, Articles 126–130).

2 The JEP may summon former FARC-EP members, state agents belonging to the security forces, and third parties, including civilians, who have been prosecuted for or committed crimes related to the armed conflict.

3 For more on Macrocases, see: https://www.jep.gov.co/Paginas/casos.aspx.

4 In this regard, the JEP has approved several documents. For instance: Guidelines for the Implementation of the Ethnic-Racial Approach. Available https://acortar.link/IBQGsd.

5 See Truth, Coexistence and Non-Repetition Commission (2022, p. 319, 582 y 583).

6 The PDET is a subregional management tool for the comprehensive transformation of rural areas, implemented in the territories most affected by the armed conflict. For more information, see: https://centralpdet.renovacionterritorio.gov.co/que-es-el-pdet/.

7 Macrocase 06, “Victimization of Members of the Patriotic Union (UP),” reflects the recognition of non-ethnic collective victims.

8 This process is called dialogic procedure (Cote Barco, 2020), and it is a setting that promotes dialogue, deliberation, and the collective construction of truth among the victims, the JEP and the offenders (Special Jurisdiction for Peace, 2024b, p. 34).

9 The JEP has approved several documents. For instance: Protocol No. 001 of 2019. Available https://www.jep.gov.co/DocumentosJEPWP/protocolo.pdf.

10 JEP, SRVR, Auto No. 078/2018, 8 November 2018, par. 10.

11 Indigenous and Afro-descendant communities seek recognition of collective territories and autonomous forms of administration and governance. Indigenous resguardos and cabildos, as well as Afro-descendant collective territories and community councils, embody these forms of self-government.

12 JEP, Auto SRVR No. 001 de 2023, Auto de determinación de hechos y conductas dentro del Caso No. 05, par. 505, 506.

13 JEP, Auto SRVR No. 001 de 2023, Auto de determinación de hechos y conductas dentro del Caso No. 05, par. 510. 511.

14 JEP, Auto SRVR No. 02 de 2024, Segundo Auto de Determinación de Hechos y Conductas dentro del Caso No. 05, par. 634.

15 JEP, SRVR, Auto No. 078/2018, 8 November 2018, par. 535.

16 JEP, Auto SRVR No. 001 de 2023, par. 534.

17 For damages to the cultural identity of the Nasa people see (Cote Barco and Vega Dueñas, 2022).

18 JEP, Auto SRVR No. 001 de 2023, par. 328–533.

19 JEP, SRVR-02/2020, 17 January 2020, Accreditation of the CRIC and the territory Chab Wala Kiwe, par. 14.

20 JEP, Auto 226/2023, 11 July 2023, Accreditation Cauca River, par. 42.

21 The public hearing on the acknowledgment of truth and responsibility constitutes the procedural stage during which offenders appearing before the JEP formally acknowledge responsibility for the facts and conduct attributed to them following the evidentiary assessment and corroboration process (Special Jurisdiction for Peace, 2024b, p. 165).

22 Cauca Regional Indigenous Council (CRIC), Changual Aquiwe Asín; Satta Maquiwe and UG Walavits territories; reserves and community councils of Valle del Cauca; Oribc and Nazüay organizations; women weavers and women searchers from the Satamaquiwe territory.

23 Association of Community Councils of Northern Cauca (ACONC); multiple community councils from Pradera and Florida; Association of Women of African Descent of Northern Cauca (ASOM); Buenos Aires Victims’ Roundtable; various organizations grouped under Afroamérica Siglo XXI.

24 Community Action Boards of Florida, the Municipal Association of Peasant Farmers of Pradera (AMUC), Fensuagro, Asposonac (Miranda Peasant Reserve), and the Association of Families of Victims of the Armed Conflict in Caldono (Asfabical).

25 Through this decision, the Chamber for the Recognition of Truth identifies the established facts and conduct, the persons responsible, and the type of responsibility attributed to them.
